# Integrating single-nucleus sequence profiling to reveal the transcriptional dynamics of Alzheimer’s disease, Parkinson’s disease, and multiple sclerosis

**DOI:** 10.1186/s12967-023-04516-6

**Published:** 2023-09-21

**Authors:** Li-Yuan Fan, Jing Yang, Ruo-Yu Liu, Ying Kong, Guang-Yu Guo, Yu-Ming Xu

**Affiliations:** 1 Department of Neurology, First Affiliated Hospital of Zhengzhou University, Zhengzhou University, Zhengzhou, China; 2 Academy of Medical Sciences of Zhengzhou University, Zhengzhou, China

**Keywords:** Alzheimer’s diseases, Parkinson’s disease, Multiple sclerosis, Single-cell sequence, Crosstalk, *HSPB1*, *HSPA1A*, Ribosomal proteins, Arctigenin

## Abstract

**Background:**

Alzheimer's disease (AD), Parkinson's disease (PD), and multiple sclerosis (MS) are three nervous system diseases that partially overlap clinically and genetically. However, bulk RNA-sequencing did not accurately detect the core pathogenic molecules in them. The availability of high-quality single cell RNA-sequencing data of post-mortem brain collections permits the generation of a large-scale gene expression in different cells in human brain, focusing on the molecular features and relationships between diseases and genes. We integrated single-nucleus RNA-sequencing (snRNA-seq) datasets of human brains with AD, PD, and MS to identify transcriptomic commonalities and distinctions among them.

**Methods:**

The snRNA-seq datasets were downloaded from Gene Expression Omnibus (GEO) database. The Seurat package was used for snRNA-seq data processing. The uniform manifold approximation and projection (UMAP) were utilized for cluster identification. The FindMarker function in Seurat was used to identify the differently expressed genes. Functional enrichment analysis was carried out using the Gene Set Enrichment Analysis (GSEA) and Gene ontology (GO). The protein‐protein interaction (PPI) analysis of differentially expressed genes (DEGs) was analyzed using STRING database (http://string-db.org). SCENIC analysis was performed using utilizing pySCENIC (v0.10.0) based on the hg19-tss-centered-10 kb-10species databases. The analysis of potential therapeutic drugs was analyzed on Connectivity Map (https://clue.io).

**Results:**

The gene regulatory network analysis identified several hub genes regulated in AD, PD, and MS, in which *HSPB1* and *HSPA1A* were key molecules. These upregulated HSP family genes interact with ribosome genes in AD and MS, and with immunomodulatory genes in PD. We further identified several transcriptional regulators (*SPI1*, *CEBPA*, *TFE3*, *GRHPR*, and *TP53*) of the hub genes, which has important implications for uncovering the molecular crosstalk among AD, PD, and MS. Arctigenin was identified as a potential therapeutic drug for AD, PD, and MS.

**Conclusions:**

Together, the integrated snRNA-seq data and findings have significant implications for unraveling the shared and unique molecular crosstalk among AD, PD, and MS. *HSPB1* and *HSPA1A* as promising targets involved in the pathological mechanisms of neurodegenerative diseases. Additionally, the identification of arctigenin as a potential therapeutic drug for AD, PD, and MS further highlights its potential in treating these neurological disorders. These discoveries lay the groundwork for future research and interventions to enhance our understanding and treatment of AD, PD, and MS.

**Supplementary Information:**

The online version contains supplementary material available at 10.1186/s12967-023-04516-6.

## Introduction

In face of aging world population and the absence of effective cures, central nervous system (CNS) diseases pose a significant economic burden on society. Alzheimer’s disease (AD), Parkinson’s disease (PD), and multiple sclerosis (MS) are chronic and progressive diseases of the CNS, characterized by the loss of neurons in the brain or the spinal cord [1]. Although a variety of efforts at the molecular level have attempted to elucidate the basic biological pathologies contributing to these diseases, the principal causes and cures for these diseases remain elusive, which hinders the discovery of disease-modifying therapies.

AD is the most common neurodegenerative disease, identified by the presence of extracellular aggregates of amyloid β (Aβ) peptides and intraneuronal tau neurofibrillary tangles in the brain. Patients with AD primarily exhibit the impairments in short-term memory and cognitive processing [2]. PD is the second most common neurodegenerative disease, and is clinically defined by the symptoms of akinesia, rigidity, and tremor. Cytosolic Lewy bodies aggregated by α-synuclein and the loss of dopaminergic neurons in the substantia nigra pars compacta are the major neuropathological features [3]. MS is a progressive neuroinflammatory disease with distinct lesion characteristics in the cortical grey versus subcortical white matter, and neurodegeneration at chronic stages. Clinically, the symptoms of MS are diverse, involving the impairments of movement, sensation, vision, and cognition [4]. Although AD, PD, and MS differ in many clinical and pathological aspects, it is possible that they share cross-molecular features. Growing evidence indicates that brain endothelial dysfunction might play a significant role in the neurobiology of AD, PD, and MS [5, 6], which causes derangement of the mitochondrial machinery [7], suppressing glutamate reuptake by astrocytes and resulting in glutamate-mediated toxicity [8]. Immune cell infiltration from the periphery into the CNS as well as the inflammatory responses mediated by reactive astrocytes and by activated microglia in the CNS also have been implicated in AD, PD, and MS [9]. Elucidating the shared and distinct molecular crosstalk could provide a biological basis for the treatments of AD, PD, and MS, which remains controversial in the field.

The establishment of single-nucleus RNA-sequencing (snRNA-seq) databases over the last decade permits global bioinformatics analyses of gene expression in different cells. Transcriptomic profiling, through snRNA-seq of patient-derived tissues, can address confounding by cellular composition, providing previously unattainable insight into cell-type-specific transcriptomic pathology [10, 11]. Existing data resources have not yet been fully exploited to understand the causal disease pathways in AD, PD, and MS. With this in mind, we integrated several existing human snRNA-seq datasets to gain a comprehensive view of the cell-type-specific transcriptional changes of these diseases in the CNS. Our results showed the shared and distinct transcriptional changes in multiple cell types among AD, PD, and MS. We hypothesized that *HSPB1* may be the core molecule of the shared pathological mechanism of the three diseases, which is the result of the induction by the blood–brain barrier (BBB) in response to cellular stress, providing insight into the nervous system diseases with unique pathogenic processes. Additionally, arctigenin has shown potential as a therapeutic drug for AD, PD, and MS.

## Methods

### Resources of single-nucleus RNA sequencing data

The selection of datasets was driven by the specific disease contexts and the relevant anatomical sites, which mainly based on the specific disease pathology. The complete snRNA-seq data sets used in this study were downloaded from the NCBI Gene Expression Omnibus database (GEO; https://www.ncbi.nlm.nih.gov/geo/) under accession numbers GSE138852 [12], GSE174367 [13], GSE157783 [14], and GSE118257 [15] (Table 1; Additional file 2: Table S1). Dataset GSE138852 contains entorhinal cortex samples from control and AD brains (n = 6 per group). GSE174367 contains prefrontal cortex samples from control (n = 8) and late-stage AD brains (n = 12). GSE157783 contains midbrain samples from idiopathic PD (n = 5) and control brains (n = 6). GSE118257 contains white matter samples from patients with MS (n = 12) and controls (n = 9).

**Table 1:** Summary of snRNA-seq datasets used in this study

Accession ID	Disease	Year	Data type	Brain region	Nucleus number	Controls/Cases
GSE138852	AD	2019	snRNA-seq	Entorhinal cortex	12,777	6/6
GSE174367	AD	2021	snRNA-seq	Prefrontal cortex	58,894	8/12
GSE157783	PD	2022	snRNA-seq	Midbrain	41,377	5/6
GSE118257	MS	2019	snRNA-seq	White matter	16,778	16/17

The nucleus number were the amounts of the nuclei after the quality control

### Quality control, normalization, batch correction, dimensionality reduction, and clustering

All bioinformatic analyses were performed with Seurat (version 4.0; https://satijalab.org/seurat/) [16] in R software (version 4.0.2) for data processing and analysis. We obtained the gene expression matrix and metadata information of GSE138852, GSE174367, and GSE118257 datasets from GEO database. The metadata information includes cell-specific details such as cell origin, tissue source, disease status, and other relevant annotations. The expression matrix was directly transformed into a Seurat object using the CreateSeuratObject() function. Subsequently, we systematically incorporated the metadata information into this Seurat object. For the GSE157783 dataset, we downloaded three files from GEO, namely the cell barcode file, gene feature file, and expression matrix file. To integrate these files, we employed the Read10X() function, which resulted in an expression matrix with genes represented as rows and cells as columns. Then, the CreateSeuratObject() function was employed to generate a Seurat object. Finally, we utilized the merge function to integrate four Seurat objects together, facilitating further analysis. Cells with less than 200 genes, mitochondrial counts greater than 5%, and genes expressed in less than three cells were filtered out for quality control. The data was first normalized by functions NormalizeData and ScaleData functions. Then FindVariable function was applied to select the top 2000 variable genes. We conducted principal component analysis (PCA) using the top 2000 variable genes. We selected the top 10 principal components (PCs) for sub-clustering and set the resolution parameter to 0.1. To mitigate batch effects and non-biological technical biases, we employed the Harmony package in Seurat (https://github.com/immunogenomics/harmony). Default parameter values were utilized for Harmony settings. The expression of known marker genes was used as a reference for annotation of different cell types. The results were visualized by Uniform Manifold Approximation and Projection (UMAP).

### Differential expression analysis and functional enrichment analysis

To identify the genes that were upregulated in AD, PD, and MS brains across different cell types, the FindMarker function (Logfc.threshold = 1, *p* < 0.05, Only.pos = T, pct.1 − pct.2 > 0.2, Wilcoxon test) in Seurat was used. In addition, we defined the active (MS_Active) and chronic active (MS_CA) samples as the disease group to identify the truly upregulated genes in MS. Functional enrichment analysis was carried out using the Gene Set Enrichment Analysis (GSEA) [17] and hypergeometric tests with the clusterProfiler R package. Gene ontology (GO) enrichment analyses was performed using clusterProfiler R package with the input species set to Homo sapiens. The protein‐protein interaction (PPI) analysis of differentially expressed genes (DEGs) was analyzed using STRING database (http://string-db.org).

### Gene regulatory network analysis

Single-cell regulatory network inference and clustering (SCENIC) analysis was performed using utilizing pySCENIC (v0.10.0) based on the hg19-tss-centered-10 kb-10species databases (https://github.com/aertslab/pySCENIC) [18]. Default parameters were used for the SCENIC workflow, and the raw count matrix from all the samples was used as the input. A three-step process was used for the analysis. Firstly, we calculated the co-expression modules and evaluated the weight between transcriptional factors (TFs) and their target genes using GRNBoost, and then TFs with direct targets (regulons) were identified using RcisTarget. Finally, the activity of each regulon in each cell was evaluated using AUCell. For visualization, the average regulon activity (AUC) scores for each cell type were calculated, and a rank plot of regulons was drawn using ggplot2.

### Connectivity map bioinformatics analysis

The connectivity map was analyzed on Connectivity Map (https://clue.io) [19, 20] with default settings. The differentially up-regulated genes in AD, PD, and MS were uploaded to the Connectivity Map database for analysis of potential therapies with gene expression signatures. Normalized connectivity score (nomalized_cs) < 0 and *p* < 0.05 as the threshold.

## Results

### Multi-dataset integration revealed the single cell transcriptional states of AD, PD, and MS

**Fig. 1:**
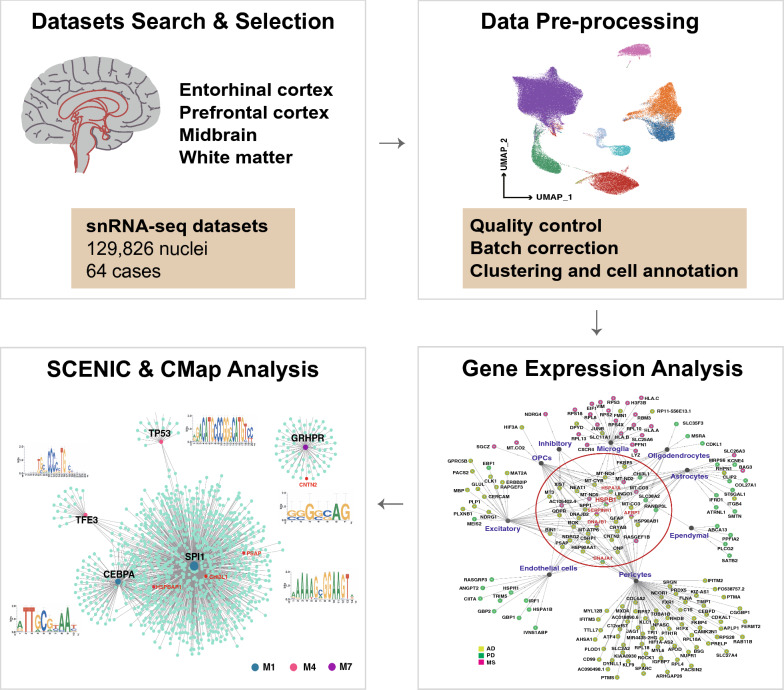
The workflow of the study. After literature search, 64 cases from different single-nucleus sequence studies were selected. These studies were done on post-mortem human brain tissue, which were categorized into entorhinal cortex, prefrontal cortex, midbrain, and white matter, severely affected regions by AD, PD, and MS pathology, respectively

**Fig. 2:**
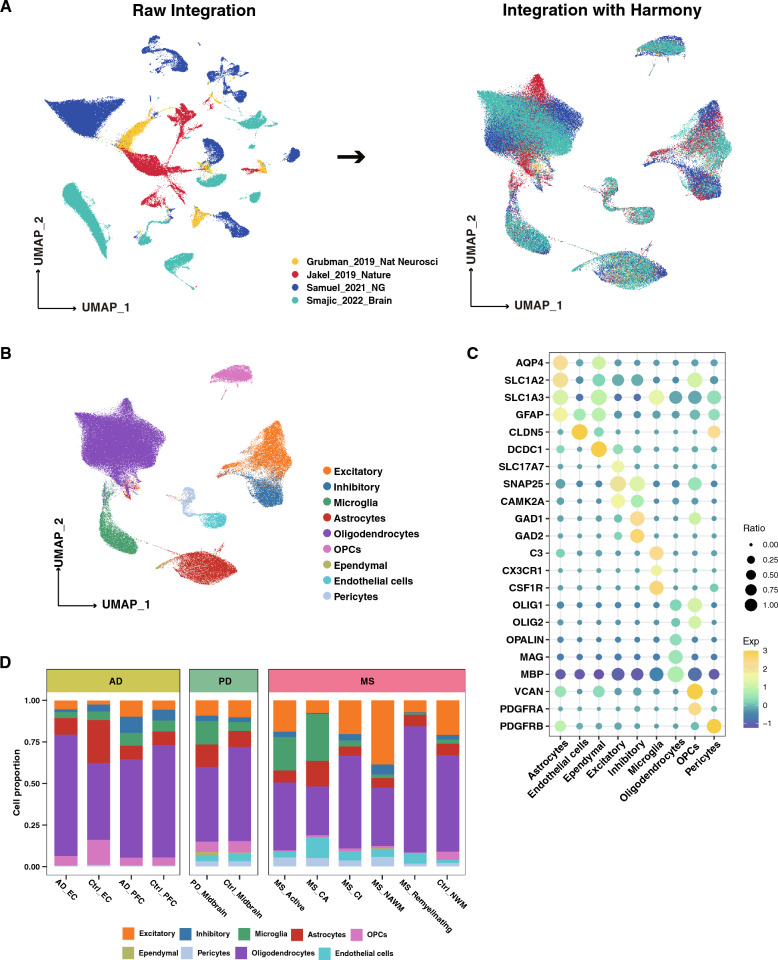
Single cell transcriptional landscape of AD, PD, and MS. **A** Uniform Manifold Approximation and Projection (UMAP) plot of the combined datasets before (right) and after (light) batch correction with Harmony, colored by dataset source. **B** UMAP representation of the landscape of different CNS cell types. **C** Dot-plots for the merged snRNA-seq data demonstrates the marker expressions in the different nuclei clusters. **D** Stacked bar plots of the differing cell-type proportions in the merged dataset. MS_Active (active lesions), MS_CA (chronic active lesions), MS_CI (chronic inactive lesions), MS_NAWM (non-lesioned, normal appearing white matter), MS_ Remyelinating (remyelinating lesions), and Ctrl_NWM (normal white matter)

To understand the shared and distinct transcriptional responses in patients with nervous diseases, we integrated human brain snRNA-seq datasets, spanning the entorhinal cortex (EC), prefrontal cortex (PFC), midbrain, and white matter from 64 cases (Fig. 1, Table 1, Additional file 2: Table S1). As a result of variations in specimen sources, experimental conditions, and library preparation methods, batch effects were observed in the dimensionally reduced visualizations of these datasets (Fig. 2A). Following the application of Harmony integration, the batch effects were effectively mitigated, with no prominent separation observed across different platforms or conditions (Fig. 2A). Unsupervised nucleus clustering, differential expression analysis, and classification were performed on the merged snRNA-seq data. In the UMAP space, we profiled a total of nine major cell types with 129,826 nuclei by employing Seurat’s data integration pipeline [21] (Fig. 2B). Cell types identified on the basic of defining markers included excitatory neurons (*CAMK2A*^+^, *SLC17A7*^+^, *SNAP25*^+^), inhibitory neurons (*GAD1*^+^, *GAD2*^+^), microglia (*C3*^+^, *CX3CR1*^+^, *CSF1R*^+^), astrocytes (*AQP4*^+^, *SLC1A2*^+^, *SLC1A3*^+^, *GFAP*^+^), oligodendrocytes (*MBP*^+^, *OLIG1*^+^, *OLIG2*^+^, *OPALIN*^+^, *MAG*^+^), oligodendrocyte precursors (OPCs) (*PDGFRA*^+^, *VCAN*^+^), ependymal cells (*DCDC1*^+^), endothelial cells (*CLDN5*^+^), and pericytes (*PDGFRB*^+^) (Fig. 2C). The UMAP plot displayed the same expression patterns of the markers (Additional file 1: Fig. S1). We estimated cell-type proportions across disease groups and observed a decrease in oligodendrocytes and a marked increase in microglia occurred in the active (MS_Active) and chronic active lesion areas (MS_CA) of MS patients, and the midbrain of PD relative to the control groups; these changes were not observed in the AD group (Fig. 2D). OPCs were also significantly reduced in the active and CA lesion areas of MS, which is consistent with demyelination in the MS brain (Fig. 2D).

### Defining potential shared gene regulatory network among AD, PD, and MS

**Fig. 3:**
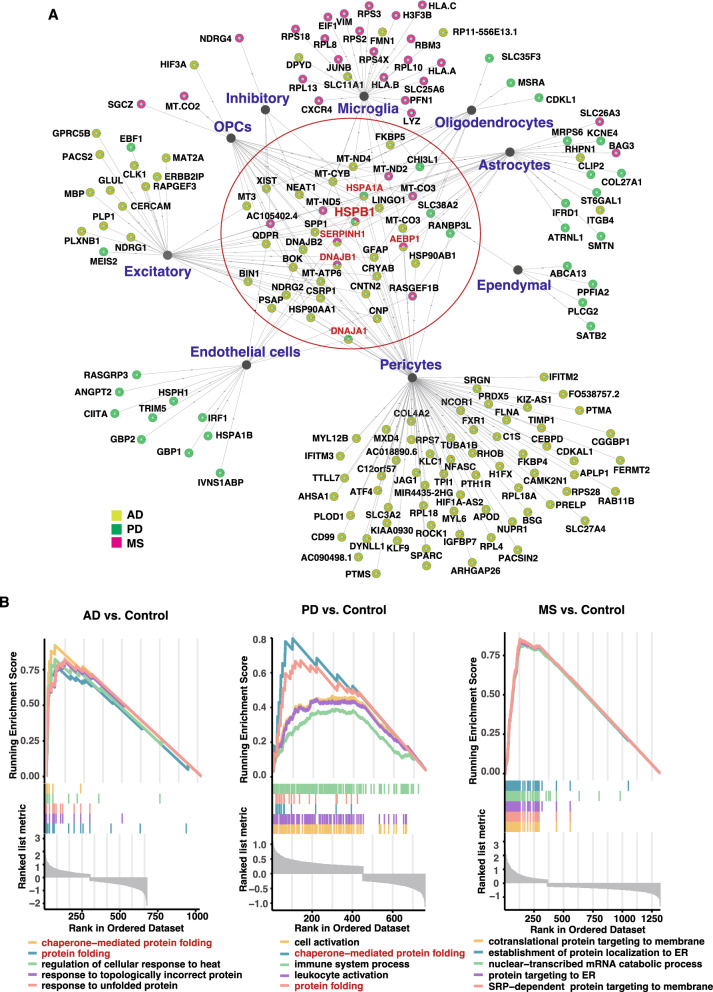
Network of DEGs and enrichment analysis. **A** The network of upregulated genes across different cell types in AD, PD, and MS. **B** Enrichment plots from Gene set enrichment analysis (GSEA), the top five biological pathways sorted by normalized enrichment score across AD (left panel), PD (middle panel) and MS (right panel) are shown. The color of broken line represents different pathways

To identify the shared gene regulatory network among AD, PD, and MS, we analyzed the association degree of DEGs (AD *vs.* control, PD *vs.* control, MS_Active and MS_CA *vs.* Ctrl_NWM) connectivity among the three diseases (Fig. 3A, Additional file 3: Table S2, Additional file 4: Table S3, Additional file 5: Table S4). Most genes upregulated in AD were expressed in excitatory neurons, astrocytes, and pericytes (Fig. 3A, Additional file 3: Table S2). PD-associated upregulated genes were found in almost all endothelial cells and astrocytes (Fig. 3A, Additional file 4: Table S3). Most of the upregulated genes in MS were found in endothelial cells, microglia, OPCs, and astrocytes (Fig. 3A, Additional file 5: Table S4). Upregulated DEGs that shared at least two cell types among three diseases were defined as hub-genes (Fig. 3A). Interestingly, the hub gene *HSPB1* was identified to be significantly upregulated in the astrocytes, pericytes, OPCs, and excitatory cells of AD brains, and in endothelial cells of PD and MS brains (Fig. 3A, Additional file 3: Table S2, Additional file 4: Table S3, Additional file 5: Table S4). *HSPA1A* was found upregulated in oligodendrocytes, OPCs, pericytes, astrocytes, and excitatory cells of AD brains, and of oligodendrocytes, pericytes, and endothelial cells of PD brains (Fig. 3A, Additional file 3: Table S2, Additional file 4: Table S3). *DNAJA1* was upregulated in pericytes of AD and in endothelial cells of PD (Fig. 3A, Additional file 3: Table S2, Additional file 4: Table S3). Additionally, *SERPINH1*, *AEBP1*, and *DNAJB1* were similarly upregulated in both AD and MS (Fig. 3A, Additional file 3: Table S2, Additional file 5: Table S4).

To summarize the integrated ranking in the pathway, we performed GO analysis using GSEA (Fig. 3B). We prioritized the signaling pathways and identified five major pathways in each disease (*p* < 0.05, normalized enrichment score (NES) > 0, Fig. 3B). In the line part, the horizontal axis is the sorted gene, and the vertical axis is the corresponding Running Enrichment Score. There is a peak in the line chart, which is the Enrichment score of this gene set, and the corresponding gene is the core of the gene set. Collectively, two common pathways were identified in AD and PD: chaperone-mediated protein folding (GO: 0061077) and protein folding (GO: 0006457) (Fig. 3B), consistent with the well-established pathological mechanism of protein folding abnormalities in AD and PD [22]. Interestingly, MS-related biological processes were mainly related to the transport of proteins to the endoplasmic reticulum (ER) (GO: 0006613, GO: 0072599, GO: 0045047, GO: 0006614) (Fig. 3B).

**Fig. 4:**
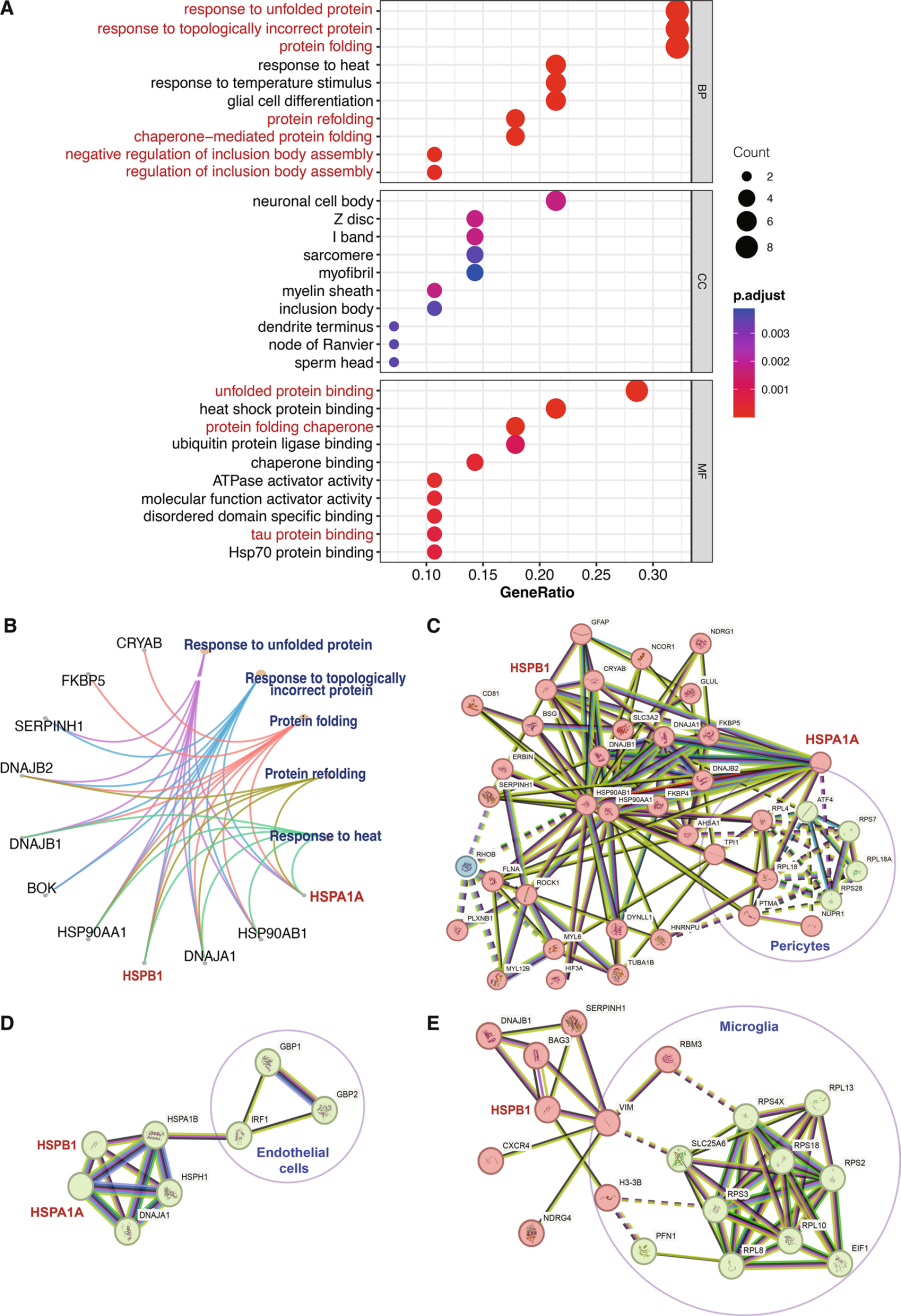
Functional enrichment analysis of differentially expressed genes among three diseases. **A** Gene ontology (GO) functional enrichment analyze based on the hub genes. BP: biological process. CC: cellular components. MF: molecular function. **B** Network visualization of relationships of enriched functions and genes. **C** Protein–protein interaction (PPI) network of differentially expressed genes (DEGs) in AD brains. **D** PPI network of DEGs in PD brains. **E** PPI network of DEGs in MS brains

Both GO biological process and molecular functions analysis of the hub-genes revealed a significant enrichment in the protein folding and regulation of inclusion body assembly, which may module of the process of the pathological protein aggregation (Fig. 4A). *HSPB1* and *HSPA1A* were both involved in multiple protein folding-related biological processes (Fig. 4B). STRING analysis of DEGs has unveiled the significant involvement of the heat shock protein (HSP) family in the pathogenesis of neurodegenerative diseases. In the brains of individuals with AD, *HSPB1* and *HSPA1A* interacted with *DNAJB1* in pericytes and excitatory cells, and with *DNAJA1* in pericytes (Fig. 4C, Additional file 3: Table S2). In PD, *HSPB1* and *HSPA1A* interacted with *DNAJA1* in endothelial cells (Fig. 4D, Additional file 4: Table S3). Moreover, in the brains of individuals with MS, *HSPB1* interacted with *DNAJB1* in endothelial cells (Fig. 4E, Additional file 5: Table S4). We simultaneously observed that a series of ribosomal proteins play important roles in the pericytes of AD brains (Fig. 4C). For instance, *RPL4*, *RPS7*, *RPL18*, *RPL18A*, and *RPS28* were all upregulated in the pericytes (Fig. 4C, Additional file 3: Table S2). These ribosomal proteins also interact directly or indirectly with *HSPA1A* (Fig. 4C). In PD, the HSP family interacted with *IRF1* in endothelial cells, potentially mediating the regulation of inflammatory responses (Fig. 4D, Additional file 4: Table S3). In MS, *HSPB1* not only demonstrated its role in upregulated proteins of astrocytes but also exhibited close interactions with upregulated ribosomal proteins of microglia (Fig. 4E, Additional file 5: Table S4).

### Transcriptional dynamics and the regulators of AD, PD, and MS

**Fig. 5:**
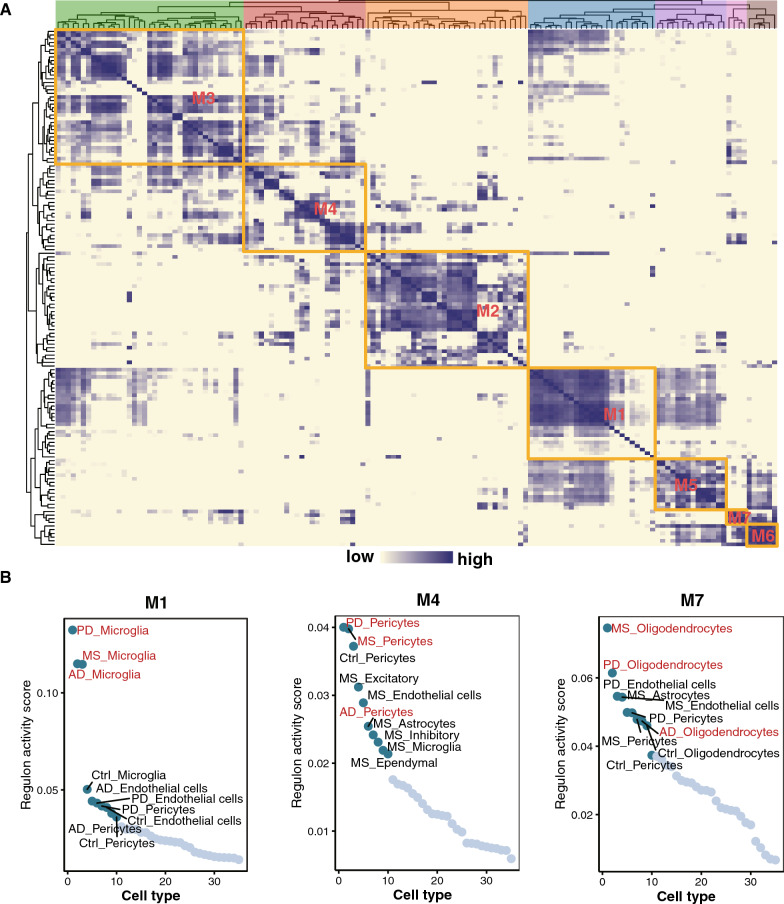
SCENIC analysis showing distinct and shared regulons across different cell types. **A** Heatmap of the identified TFs with hierarchical clustering. Different colors represent different modules. **B** Regulons activity scores of different cell types among modules (M1, M4, and M7)

The SCENIC was used to map the gene regulatory networks governing the different diseases and identify potential TFs modulating the DEGs in disease samples. We initially identified the modules of TFs which regulate the cell heterogeneity (Fig. 5A, Additional file 6: Table S5). Some modules of TFs were highly specific in AD, PD, and MS (M1, M4, and M7) (Fig. 5B, Additional file 6: Table S5). Using the regulon activity scores (RAS), we identified some regulons that were active in specific cell types among three diseases, regulating cell type-specific functions (Fig. 5B). Regulons activity in the M1 module was higher in the microglia of among three diseases (Fig. 5B). In the M4 module, the active regulons were mostly found in pericytes, especially in patients with PD and MS (Fig. 5B). Most of regulons in the M7 module were in the oligodendrocytes of PD and MS patients, indicating that PD and MS patients may share highly active TFs (Fig. 5B). Information regarding the modules and regulons is provided in Additional file 6: Table S5.

**Fig. 6:**
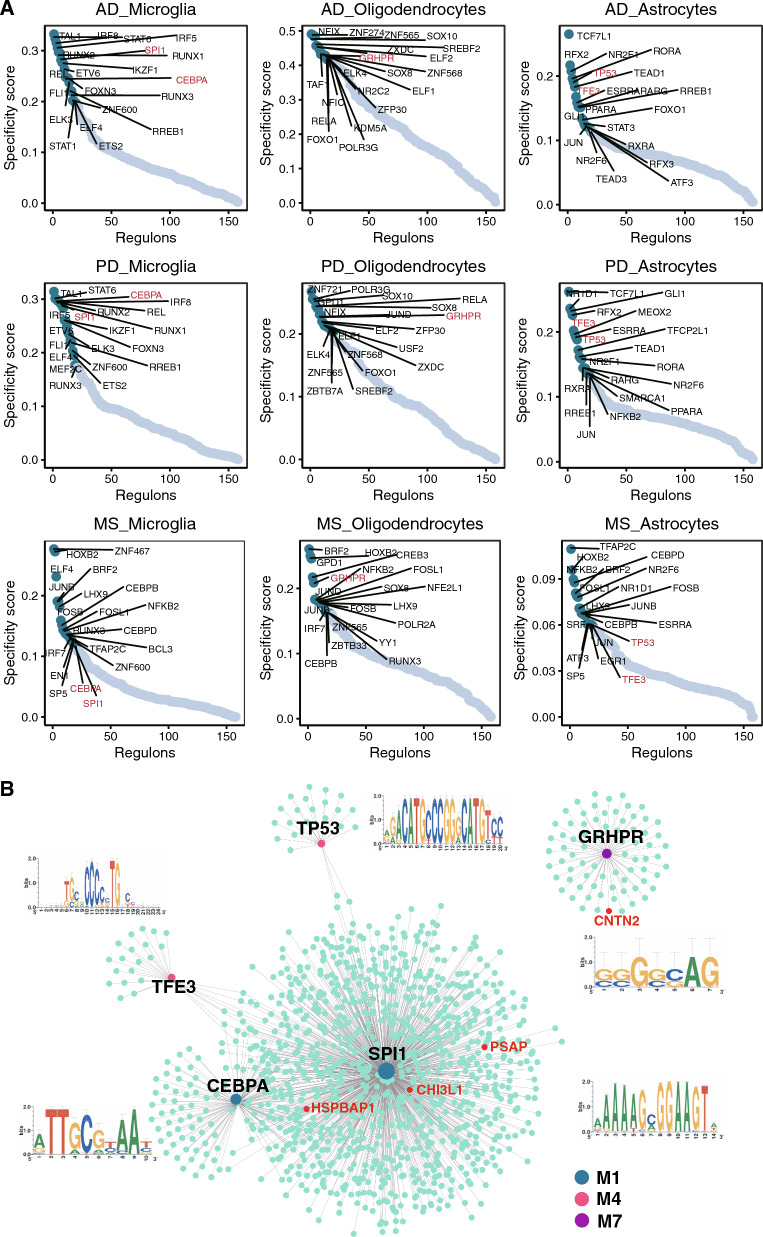
Transcriptional regulatory network among AD, PD, and MS. **A** Specificity scores of regulons in microglia, oligodendrocytes, and astrocytes. The top 20 genes with higher activity are noted. **B** Network of TFs shared by the three diseases and their target genes (regulons), accompanied by the corresponding motifs of the TFs

To obtain a specific correspondence between regulons and each cell type, we further analyzed the top 20 specific regulons in different cell types using the regulon specificity score (RSS) (Fig. 6A, Additional file 7: Table S6). We identified some shared specific regulons among three diseases by the Specificity score as candidate transcription factors underlying the gene expression differences in different cells (Fig. 6A). *CEBPA* and *SPI1* in microglia, *GRHPR* in oligodendrocytes, and *TFE3* and *TP53* in astrocytes were shared by AD, PD, and MS (Fig. 6A). By integrating the transcription profiles previously identified upregulated in the three disease groups, we conducted an analysis to identify the candidate target genes regulated by these shared TFs (Fig. 6B). We identified several target genes that showed up-regulation in the disease groups, including *PSAP* and *CNTN2* in AD, as well as *CHI3L1* in PD (Fig. 6B, Additional file 8: Table S7). Interestingly, *HSPBAP1*, which interacts with hub-gene *HSPB1* [23], was identified as a target gene regulated by both *CEBPA* and *SPI1* (Fig. 6B). The corresponding motifs are showed in Fig. 6B. These analyses identified the upstream regulons that drive cell-type-specific state transitions toward disease.

### Discovery of repurposable drugs

**Fig. 7:**
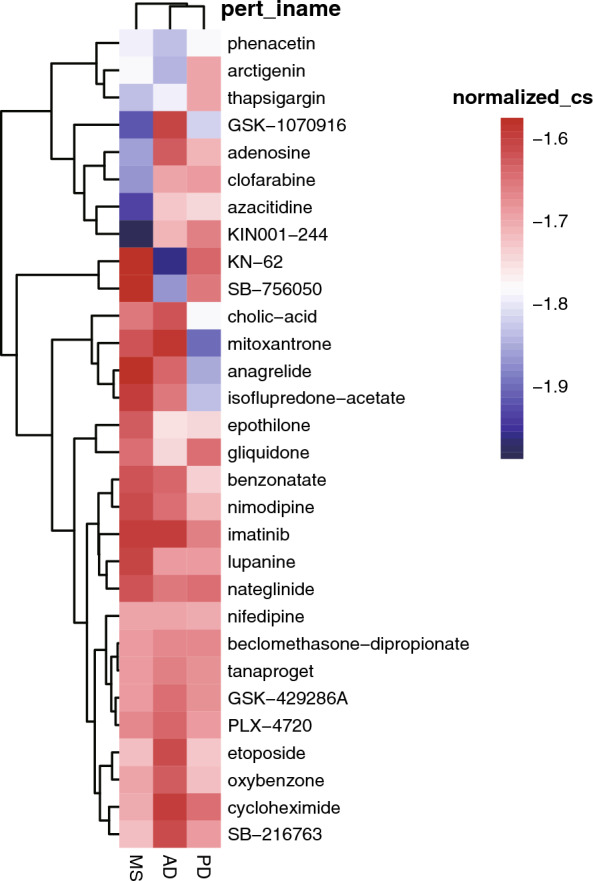
The shared 30 therapeutic drugs of AD, PD, and MS. Drug sensitivity analyses were performed using Connectivity Map (CMap). Threshold normalized connectivity score (normalized_cs) was set at < 0, and *p* < 0.05

Connectivity Map (CMap) (https://clue.io) [20] is a genome-wide transcriptional expression dataset of selected human cell lines processed by bioactive small molecules, including many drugs. CMap is used to discover functional links between drugs, genes, and diseases through transient signatures of common gene expression changes. With CMap, we identified that 30 shared drugs were inversely correlated with AD, PD, and MS (Fig. 7, Additional file 9: Table S8, Additional file 10: Table S9, Additional file 11: Table S10). Calcium channel blocker, topoisomerase inhibitor, MEK inhibitor, DNA methyltransferase inhibitor, and adenosine receptor agonist were inversely correlated with the up-regulated genes in AD, PD, and MS (Table 2). Arctigenin is a phenylpropanoid dibenzylbutyro lactone lignan compound which showed a potential therapeutic agent for AD, PD, and MS in our study (Fig. 7). Arctigenin has been shown having neuroprotective effects in vivo and in vitro of AD models [24]. Previous studies showed that arctigenin effectively ameliorated memory impairment in AD mice model by targeting the production and clearance of β-amyloid [25]. Arctigenin could improve the movement behaviors and upregulate dopamine and γ-aminobutyric acid levels in a PD mice model [26]. Arctigenin may have anti-inflammatory and immunosuppressive properties via inhibiting Th17 cells in MS [27]. These findings suggested that arctigenin holds promise as a potential therapeutic agent for AD, PD, and MS.

**Table 2:** The names, Moa, and targets of the 30 drugs

Drug name	Moa	Target name
Adenosine	Adenosine receptor agonist	ADORA1|ADORA2A|ADORA2B|ADORA3|PI4K2A|PI4K2B|TRPM4
Anagrelide	Phosphodiesterase inhibitor	PDE3A
Arctigenin	MEK inhibitor	ADIPOR1|AHR|CHUK|MAP2K1
Azacitidine	DNA inhibitor	DNMT1|DNMT3A
Azacitidine	DNA methyltransferase inhibitor	DNMT1|DNMT3A
Beclomethasone-dipropionate	Immunosuppressant|Glucocorticoid receptor agonist	NR3C1|CYP3A5|ADGRG3
Benzonatate	Anesthetic—local	SCN5A
Cholic-acid	Bile acid	CES1|FECH|PLA2G1B|ADH1C|COX1|COX2|COX3|COX4I1|COX5A|COX5B|COX6A2|COX6B1|COX6C|COX7A1|COX7B|COX7C|COX8A|ESRRG|FABP6|GPBAR1
Clofarabine	Ribonucleoside reductase inhibitor	RRM1|POLA1|RRM2|SLC22A8|POLD1|POLE|RRM2B
Cycloheximide	Protein synthesis inhibitor	GSK3B|RPL3
Epothilone	Tubulin inhibitor	TUBB
Etoposide	Topoisomerase inhibitor	TOP2A|TOP2B|CYP2E1|CYP3A5
Gliquidone	Sulfonylurea	ABCC8|KCNJ11|KCNJ10|KCNJ8
GSK-1070916	Aurora kinase inhibitor	AURKB|AURKC|AURKA|CYP2D6|CYP3A4
GSK-429286A	Rho associated kinase inhibitor	ROCK1
Imatinib	PDGFR inhibitor|Bcr-Abl inhibitor|KIT inhibitor	ABL1|KIT|PDGFRA|BCR|CSF1R|PDGFRB|ABCG2|CYP2C19|CYP2C8|CYP3A5|DDR1|NTRK1|RET
Isoflupredone-acetate	Glucocorticoid receptor agonist	NR3C1
KIN001-244	PDK1 Inhibitor	PDPK1
KN-62	Calcium channel blocker|Purinergic receptor antagonist	P2RX7|CAMK2A|AKT1|CHEK1|LCK|MAPK1|MAPK11|MAPK12|MAPK14|MAPK8|PRKCA|ROCK1|RPS6KB1|SGK1
Lupanine	Sodium channel inhibitor	INS
Mitoxantrone	Topoisomerase inhibitor	TOP2A|PIM1
Nateglinide	Insulin secretagogue	ABCC8|KCNJ11|CYP3A4|INS|KCNJ10|PPARG
Nifedipine	Calcium channel blocker	CACNA1C|CACNA1D|CACNA1S|CACNA1F|CACNA2D1|CYP3A5|CACNA1H|CACNB2|CALM1|GLRA1|GLRA3|GLRB|KCNA1|KCNA5|NR1I2|TRPM3
Nimodipine	Calcium channel blocker	CACNA1C|NR3C2|CACNA1D|CACNA1F|CACNA1S|AHR|CACNB1|CACNB2|CACNB3|CACNB4|CFTR
Oxybenzone	Lipase inhibitor	LIPE
Phenacetin	Cyclooxygenase inhibitor	PTGS1
PLX-4720	RAF inhibitor	BRAF|KDR
SB-216763	GSK inhibitor	GSK3B|CCNA2|CDK2|GSK3A
SB-756050	G protein-coupled receptor agonist	GPBAR1
Tanaproget	Progesterone receptor agonist	PGR

## Discussion

AD, PD, and MS are CNS diseases that differ clinically. Previous studies have noted the importance of the common pathological changes in these diseases, such as small vessel disease [5] and inflammation [9]. However, the molecular crosstalk between these three diseases remains largely unknown. This study aimed to identify the key molecules which regulate the pathogenic pathways in AD, PD, and MS.

We observed that the dataset obtained from the white matter of MS patients had a higher proportion of glial cells compared to neuronal populations. The nature of the tissue and the inherent heterogeneity of the cell type present pose challenges in achieving a completely unbiased representation. The result of the higher proportion of glial populations may lead to an overemphasis on glial-specific gene expression patterns while potentially masking or downplaying certain neuronal-specific signals. Furthermore, the altered proportions in the dataset could impact the functional interpretation of the results. It is important to consider the potential contributions of glial cells to the observed gene expression patterns and their implications for the underlying biological processes.

There was significant overlap in differentially expressed genes and pathways between AD and PD in our study. In the GSEA analysis, the genes of AD and PD were co-enriched into two identical pathways, chaperone-mediated protein folding and protein folding, which has previously been shown to be associated with the abnormal protein folding in AD and PD. In our study, we found that *HSPA1A*, which encodes the major heat shock protein in the HSP70 family, was upregulated in certain cell types, including oligodendrocytes, OPCs, pericytes, and astrocytes in AD, as well as oligodendrocytes, pericytes, and endothelial cells in PD. Our findings align with prior research on AD and PD. Some studies showed that HSPA1A is upregulated in human entorhinal cortex samples and layer III pyramidal cells of AD, contributing to protein folding abnormalities, and altered synaptic transmission [28, 29]. A proteomics analysis of extracellular vesicles isolated from cerebrospinal fluid in AD also revealed elevated expression of HSPA1A compared to the mild cognitive impairment and control groups [30]. Single-cell transcriptomics also uncovered the upregulation of HSPA1A (HSP70) in endothelial cells of patients with PD, which was further confirmed in PD patients’ blood specimens and peripheral blood mononuclear cells [31, 32]. Furthermore, HSPA1A protein regulates the processing and generation of APP (amyloid precursor protein) and the production and aggregation of Aβ [33, 34]. HSPA1A exerted a significant restorative effect on neuronal morphology and functional status in the temporal cortex and hippocampal region in transgenic mouse [35]. The upregulation of HSPA1A can also play a preventive and decelerating role in PD-like neurodegeneration through its chaperone activity, which effectively inhibits α-synuclein aggregation and microglia activation [36, 37]. Taken together, our findings strongly support that HSPA1A could serve as a potential therapeutic target for AD and PD.

In our study, MS-related biological processes were mainly related to the transport of proteins to the endoplasmic reticulum (ER) (GO:0006613, GO:0072599, GO:0045047, GO:0006614), which is responsible for the proper folding and processing of polypeptide chains into functional proteins in cells. ER stress occurs when misfolded or unfolded proteins accumulate in the ER due to exogenous or endogenous factors. In MS, the destruction of the BBB leads to an escalation of pro-inflammatory damage, leading to cell damage. This is followed by oxidative damage and ER stress [38], leading to apoptosis or repair of CNS cells. These results are largely controlled by the unfolded protein response [39], which is consistent with our hypothesis. The preliminary findings indicate a strong association between ER function and MS, highlighting the need for additional investigation in this area.

The most prominent discovery arising from our analysis was the consistent upregulation of *HSPB1* (*HSP27*) across among AD, PD, and MS. An increase of HSPB1 protein in the chronic active lesions of MS brains was reported [40], as we found in the present study. HSPB1 phosphorylation also was reported to be elevated in MS and AD [41, 42]. *HSPB1* codes for a small heat shock protein that probably maintains folding competence in denatured proteins [43, 44]. In response to environmental stresses such as heat shock, HSPB1 protein is upregulated [45]. The molecular chaperone activity of HSPB1 may regulate a broad spectrum of biological processes, including the phosphorylation of neurofilament proteins and their transport along axons [46]. In AD, amyloid plaques and neurofibrillary tangles (composed of hyperphosphorylated tau) are present extracellularly and intracellularly, respectively [47]. A transgenic mouse model of AD showed HSPB1 localization in plaques [48]. By crossing the APPswe/PS1dE9 mouse model with the mouse model of HSPB1 overexpressed, spatial learning and electrophysiological parameters improved significant lye [49]. HSPB1 can delay, but not prevent fibril formation by interacting with hyperphosphorylated tau [50]; this interaction is enhanced with increased tau phosphorylation [51]. Moreover, overexpression of HSPB1 reduces the cellular deposition and cytotoxicity of α-synuclein in PD [52]. HSPB1 binds to the surface of α-synuclein fibrils, thereby reducing their hydrophobicity [53]. Based on the cumulative evidence from previous studies, we put forth the hypothesis that the observed upregulation of HSPB1 in our study strongly suggests its protective function in mitigating the formation of pathological protein aggregates, particularly in response to toxic stimuli or stressful conditions associated with neurological disorders.

We observed that the upregulation of *HSPB1* occurred in different cells in the three diseases. *HSPB1* exhibited upregulation in endothelial cells in PD and MS, as well as in astrocytes, pericytes, OPCs, and excitatory cells in AD. Initially, we attributed it to a non-specific cellular response. However, we have observed that the upregulation of *HSPB1* is consistently present in the constituent cells of the BBB, including endothelial cells, pericytes, and astrocytes, across AD, PD, and MS. This finding indicates that HSPB1 plays a role in the disruption of the BBB in three diseases. A previous study suggested that the endothelium-targeted overexpression of HSPB1 ameliorated BBB disruption after ischemic brain injury [54, 55]. The immune response of HSPB1 was also found to be present in the temporal cortex of patients with epilepsy and was mainly confined to vascular walls and glial cells [56]. As a selective physical barrier, the BBB plays a protective role in maintaining the environmental balance in the brain. One hypothesis we consider is that conceptual BBB damage may play an important role in the pathogenesis of AD, PD, and MS. BBB dysfunction contributes to the onset and progression of AD [57, 58], PD [59], and MS [60] as an upstream or downstream events. However, clinical studies in this area have shown that the BBB is less pronounced in patients with AD, PD, and MS. The BBB damage is more associated with cerebrovascular disease [61]. The recent interest in AD immunotherapy has provided a reassessment of the BBB damage, as intact BBB is a potential barrier to effective treatment of anti-amyloid immunoglobulin [62]. At the same time, dysfunctional BBB may be a risk factor for immune-mediated toxicity, including autoimmune encephalitis. Another reason for revisiting the role of BBB is that the current understandings of the pathogenesis of AD, PD, and MS are incomplete. Our findings suggest that *HSPB1* is initially induced in regions where BBB is disrupted in response to cellular stress, particularly in endothelial cells and pericytes associated with blood vessels, as well as astrocytes. This endogenous response may serve as a protective mechanism against vascular and BBB injury. Further investigation is required to understand the mechanism by which HSPB1 maintains BBB structure and function.

PPI analysis of DEGs revealed that the upregulated members of the HSP family may engage in interactions with specific proteins within various cells implicated in neurodegenerative diseases. In our study, the pericytes of AD brains appeared to be a crucial focal point. HSP family genes were upregulated in pericytes and interacted with the upregulated ribosomal protein family in AD brains. Different ribosomal proteins within the ribosome bear distinct roles and functions, collaborating to ensure accurate and efficient protein synthesis. Interestingly, previous study has also reported the elevated expression of ribosomal proteins in the capillaries of AD brains, which is remarkably like what we observed in pericytes [63]. The researchers proposed that ribosomal function is augmented in the cerebral blood vessels of AD patients, leading to an aberrant protein translation network. The clinically used anti-AD drugs donepezil and tacrine were reported to inhibit ribosome biosynthesis [64]. These viewpoints suggested the significant involvement of ribosome biosynthesis in the pathogenesis of AD and the importance of it as a therapeutic target. Increased ribosome biosynthesis also occurred in the microglia of MS, which indirectly interacted with *HSPB1*. This suggests the potential involvement of microglial protein synthesis in the pathogenesis of MS. In the endothelial cells of PD, we observed a concurrent upregulation and interaction between the HSP family and the inflammation-related gene *IRF1*. *IRF1* is involved in immune responses, cell apoptosis, and tumorigenesis. Previous study has found that IRF1 protein is upregulated in α-Syn overexpressed SH-SY5Y cells and the substantia nigra of A53T α-Syn transgenic mice. Furthermore, α-Syn overexpression facilitates the translocation of IRF1 from the cytoplasm to the nucleus, mediating neuroimmune responses [65]. Our research demonstrated the upregulation of *IRF1* and HSP family genes in endothelial cells, revealing that besides immune cells, protein processing and immune-inflammatory responses in endothelial cells are also noteworthy. In this study, although we focused on the HSP family genes and their interacting genes, their prominent roles in distinct cell types appeared to vary across different diseases. This observation suggested that different cells may play diverse roles in the context of various diseases.

The transcriptional regulatory analysis identified several modules that are highly related to the pathogenesis of AD, PD, and MS, mainly in microglial, oligodendrocytes, and pericytes. We found that multiple top 20 specific TFs in microglia, oligodendrocytes, and astrocytes were shared across the AD, PD, and MS. Combined with the hub genes identified above, we found that these shared TFs can regulate multiple hub genes, including *PSAP* and *CNTN2* in AD, and *CHI3L1* in PD. The identification of these shared TFs has opened new avenues for studying their underlying pathological mechanisms.

We further identified that arctigenin could be a potential therapeutic drug for AD, PD, and MS. Arctigenin has also been shown having neuroprotective effect in vivo and in vitro of AD and PD models [24, 26]. Arctigenin could obviously attenuate the decrease of cell survival rates in SH-SY5Y cells with PD phenotypes by acting against cell apoptosis through the decrease of Bax/Bcl-2 and caspase-3, and by reducing the surplus reactive oxygen species production and downregulating the mitochondrial membrane potential [26]. These results shed light on the potential therapeutic value of arctigenin as a treatment option for AD, PD, and MS.

## Conclusions

In summary, we performed a large-scale snRNA-seq integration of diverse neurodegenerative diseases (AD, PD, and MS) from post-mortem patients. We showed that the shared and distinct molecular networks of the three diseases, which were significantly enriched for various well-known neurodegeneration-related pathobiological biological processes. *HSPB1* was identified as a key molecule in the pathogenic mechanisms of these three diseases and is associated with the BBB. Arctigenin shows promise as a potential therapeutic agent for AD, PD, and MS. Further investigation into the pathogenic mechanisms associated with *HSPB1* is required to gain a more comprehensive understanding of the involvement of the BBB in neurodegenerative diseases.

### Supplementary Information

**Additional file 1: Fig. S1.** The UMAP plots of the expression patterns of the cell markers.

**Additional file 2: Table S1.** Patient pathology and clinical history.

**Additional file 3: Table S2.** Upregulated-genes in AD.

**Additional file 4: Table S3.** Upregulated-genes in PD.

**Additional file 5: Table S4.** Upregulated-genes in MS.

**Additional file 6: Table S5.** The modules of TFs.

**Additional file 7: Table S6.** The regulon scores.

**Additional file 8: Table S7.** The regulons.

**Additional file 9: Table S8.** The Cmap results of AD.

**Additional file 10: Table S9.** The Cmap results of PD.

**Additional file 11: Table S10.** The Cmap results of MS.

## Data Availability

The raw data of the following datasets can be found at the NCBI Gene Expression Omnibus database (GEO; https://www.ncbi.nlm.nih.gov/geo/) under accession numbers GSE138852, GSE174367, GSE157783, and GSE118257. In addition, to support data sharing and reusability, all datasets generated in this study are included in the article/Supplementary Material. All statistical analyses were conducted using R software (version 4.0.2). The R script used in this research is publicly available and can be found in Github.

## References

[1] Costa V, Aprile M, Esposito R, Ciccodicola A (2013). RNA-Seq and human complex diseases: recent accomplishments and future perspectives. Eur J Hum Genet.

[2] Nebes RD, Halligan EM, Rosen J, Reynolds CF (1998). Cognitive and motor slowing in Alzheimer's disease and geriatric depression. J Int Neuropsychol Soc.

[3] Oertel W, Schulz JB (2016). Current and experimental treatments of Parkinson disease: a guide for neuroscientists. J Neurochem.

[4] Longoni G, Rocca MA, Pagani E, Riccitelli GC, Colombo B, Rodegher M, Falini A, Comi G, Filippi M (2015). Deficits in memory and visuospatial learning correlate with regional hippocampal atrophy in MS. Brain Struct Funct.

[5] Paolini Paoletti F, Simoni S, Parnetti L, Gaetani L (2021). The contribution of small vessel disease to neurodegeneration: focus on Alzheimer's disease, Parkinson's disease and multiple sclerosis. Int J Mol Sci.

[6] Lyros E, Bakogiannis C, Liu Y, Fassbender K (2014). Molecular links between endothelial dysfunction and neurodegeneration in Alzheimer's disease. Curr Alzheimer Res.

[7] Lin MT, Beal MF (2006). Mitochondrial dysfunction and oxidative stress in neurodegenerative diseases. Nature.

[8] Boycott HE, Dallas M, Boyle JP, Pearson HA, Peers C (2007). Hypoxia suppresses astrocyte glutamate transport independently of amyloid formation. Biochem Biophys Res Commun.

[9] Piancone F, La Rosa F, Marventano I, Saresella M, Clerici M (2021). The role of the inflammasome in neurodegenerative diseases. Molecules.

[10] Krishnaswami SR, Grindberg RV, Novotny M, Venepally P, Lacar B, Bhutani K, Linker SB, Pham S, Erwin JA, Miller JA (2016). Using single nuclei for RNA-seq to capture the transcriptome of postmortem neurons. Nat Protoc.

[11] Lake BB, Chen S, Sos BC, Fan J, Kaeser GE, Yung YC, Duong TE, Gao D, Chun J, Kharchenko PV (2018). Integrative single-cell analysis of transcriptional and epigenetic states in the human adult brain. Nat Biotechnol.

[12] Grubman A, Chew G, Ouyang JF, Sun G, Choo XY, McLean C, Simmons RK, Buckberry S, Vargas-Landin DB, Poppe D (2019). A single-cell atlas of entorhinal cortex from individuals with Alzheimer's disease reveals cell-type-specific gene expression regulation. Nat Neurosci.

[13] Morabito S, Miyoshi E, Michael N, Shahin S, Martini AC, Head E, Silva J, Leavy K, Perez-Rosendahl M, Swarup V (2021). Single-nucleus chromatin accessibility and transcriptomic characterization of Alzheimer's disease. Nat Genet.

[14] Smajić S, Prada-Medina CA, Landoulsi Z, Ghelfi J, Delcambre S, Dietrich C, Jarazo J, Henck J, Balachandran S, Pachchek S (2022). Single-cell sequencing of human midbrain reveals glial activation and a Parkinson-specific neuronal state. Brain.

[15] Jäkel S, Agirre E, Mendanha Falcão A, van Bruggen D, Lee KW, Knuesel I, Malhotra D, Ffrench-Constant C, Williams A, Castelo-Branco G (2019). Altered human oligodendrocyte heterogeneity in multiple sclerosis. Nature.

[16] Butler A, Hoffman P, Smibert P, Papalexi E, Satija R (2018). Integrating single-cell transcriptomic data across different conditions, technologies, and species. Nat Biotechnol.

[17] Subramanian A, Tamayo P, Mootha VK, Mukherjee S, Ebert BL, Gillette MA, Paulovich A, Pomeroy SL, Golub TR, Lander ES (2005). Gene set enrichment analysis: a knowledge-based approach for interpreting genome-wide expression profiles. Proc Natl Acad Sci U S A.

[18] Aibar S, González-Blas CB, Moerman T, Huynh-Thu VA, Imrichova H, Hulselmans G, Rambow F, Marine JC, Geurts P, Aerts J (2017). SCENIC: single-cell regulatory network inference and clustering. Nat Methods.

[19] Lamb J (2007). The Connectivity Map: a new tool for biomedical research. Nat Rev Cancer.

[20] Lamb J, Crawford ED, Peck D, Modell JW, Blat IC, Wrobel MJ, Lerner J, Brunet JP, Subramanian A, Ross KN (2006). The Connectivity Map: using gene-expression signatures to connect small molecules, genes, and disease. Science.

[21] Stuart T, Butler A, Hoffman P, Hafemeister C, Papalexi E, Mauck WM, Hao Y, Stoeckius M, Smibert P, Satija R (2019). Comprehensive integration of single-cell data. Cell.

[22] Hipp MS, Kasturi P, Hartl FU (2019). The proteostasis network and its decline in ageing. Nat Rev Mol Cell Biol.

[23] Liu C, Gilmont RR, Benndorf R, Welsh MJ (2000). Identification and characterization of a novel protein from Sertoli cells, PASS1, that associates with mammalian small stress protein hsp27. J Biol Chem.

[24] Li Y, Lan X, Wang S, Cui Y, Song S, Zhou H, Li Q, Dai L, Zhang J (2022). Serial five-membered lactone ring ions in the treatment of Alzheimer's diseases-comprehensive profiling of arctigenin metabolites and network analysis. Front Pharmacol.

[25] Zhu Z, Yan J, Jiang W, Yao XG, Chen J, Chen L, Li C, Hu L, Jiang H, Shen X (2013). Arctigenin effectively ameliorates memory impairment in Alzheimer's disease model mice targeting both β-amyloid production and clearance. J Neurosci.

[26] Li D, Liu Q, Jia D, Dou D, Wang X, Kang T (2014). Protective effect of arctigenin against MPP+ and MPTP-induced neurotoxicity. Planta Med.

[27] Li W, Zhang Z, Zhang K, Xue Z, Li Y, Zhang Z, Zhang L, Gu C, Zhang Q, Hao J (2016). Arctigenin suppress Th17 cells and ameliorates experimental autoimmune encephalomyelitis through AMPK and PPAR-γ/ROR-γt signaling. Mol Neurobiol.

[28] Chi LM, Wang X, Nan GX (2016). In silico analyses for molecular genetic mechanism and candidate genes in patients with Alzheimer's disease. Acta Neurol Belg.

[29] Dong Y, Li T, Ma Z, Zhou C, Wang X, Li J (2022). HSPA1A, HSPA2, and hspa8 are potential molecular biomarkers for prognosis among HSP70 family in Alzheimer's Disease. Dis Markers.

[30] Muraoka S, Jedrychowski MP, Yanamandra K, Ikezu S, Gygi SP, Ikezu T (2020). Proteomic profiling of extracellular vesicles derived from cerebrospinal fluid of Alzheimer's disease patients: a pilot study. Cells.

[31] Asad Samani L, Ghaedi K, Majd A, Peymani M, Etemadifar M (2023). Coordinated modification in expression levels of HSPA1A/B, DGKH, and NOTCH2 in Parkinson's patients' blood and substantia nigra as a diagnostic sign: the transcriptomes' relationship. Neurol Sci.

[32] Huang J, Liu L, Qin L, Huang H, Li X (2022). Single-cell transcriptomics uncovers cellular heterogeneity, mechanisms, and therapeutic targets for Parkinson's disease. Front Genet.

[33] Gerber H, Mosser S, Boury-Jamot B, Stumpe M, Piersigilli A, Goepfert C, Dengjel J, Albrecht U, Magara F, Fraering PC (2019). The APMAP interactome reveals new modulators of APP processing and beta-amyloid production that are altered in Alzheimer's disease. Acta Neuropathol Commun.

[34] Hussein RM, Hashem RM, Rashed LA (2015). Evaluation of the amyloid beta-GFP fusion protein as a model of amyloid beta peptides-mediated aggregation: a study of DNAJB6 chaperone. Front Mol Neurosci.

[35] Evgen'ev MB, Krasnov GS, Nesterova IV, Garbuz DG, Karpov VL, Morozov AV, Snezhkina AV, Samokhin AN, Sergeev A, Kulikov AM (2017). Molecular mechanisms underlying neuroprotective effect of intranasal administration of human Hsp70 in mouse model of Alzheimer's disease. J Alzheimers Dis.

[36] Ekimova IV, Plaksina DV, Pastukhov YF, Lapshina KV, Lazarev VF, Mikhaylova ER, Polonik SG, Pani B, Margulis BA, Guzhova IV (2018). New HSF1 inducer as a therapeutic agent in a rodent model of Parkinson's disease. Exp Neurol.

[37] Dong Z, Wolfer DP, Lipp HP, Büeler H (2005). Hsp70 gene transfer by adeno-associated virus inhibits MPTP-induced nigrostriatal degeneration in the mouse model of Parkinson disease. Mol Ther.

[38] Mháille AN, McQuaid S, Windebank A, Cunnea P, McMahon J, Samali A, FitzGerald U (2008). Increased expression of endoplasmic reticulum stress-related signaling pathway molecules in multiple sclerosis lesions. J Neuropathol Exp Neurol.

[39] Stone S, Lin W (2015). The unfolded protein response in multiple sclerosis. Front Neurosci.

[40] Peferoen LA, Gerritsen WH, Breur M, Ummenthum KM, Peferoen-Baert RM, van der Valk P, van Noort JM, Amor S (2015). Small heat shock proteins are induced during multiple sclerosis lesion development in white but not grey matter. Acta Neuropathol Commun.

[41] Dammer EB, Lee AK, Duong DM, Gearing M, Lah JJ, Levey AI, Seyfried NT (2015). Quantitative phosphoproteomics of Alzheimer's disease reveals cross-talk between kinases and small heat shock proteins. Proteomics.

[42] Kotelnikova E, Kiani NA, Messinis D, Pertsovskaya I, Pliaka V, Bernardo-Faura M, Rinas M, Vila G, Zubizarreta I, Pulido-Valdeolivas I (2019). MAPK pathway and B cells overactivation in multiple sclerosis revealed by phosphoproteomics and genomic analysis. Proc Natl Acad Sci USA.

[43] Almeida-Souza L, Goethals S, de Winter V, Dierick I, Gallardo R, Van Durme J, Irobi J, Gettemans J, Rousseau F, Schymkowitz J (2010). Increased monomerization of mutant HSPB1 leads to protein hyperactivity in Charcot-Marie-Tooth neuropathy. J Biol Chem.

[44] Rogalla T, Ehrnsperger M, Preville X, Kotlyarov A, Lutsch G, Ducasse C, Paul C, Wieske M, Arrigo AP, Buchner J (1999). Regulation of Hsp27 oligomerization, chaperone function, and protective activity against oxidative stress/tumor necrosis factor alpha by phosphorylation. J Biol Chem.

[45] Faucher C, Capdevielle J, Canal I, Ferrara P, Mazarguil H, McGuire WL, Darbon JM (1993). The 28-kDa protein whose phosphorylation is induced by protein kinase C activators in MCF-7 cells belongs to the family of low molecular mass heat shock proteins and is the estrogen-regulated 24-kDa protein. J Biol Chem.

[46] Holmgren A, Bouhy D, De Winter V, Asselbergh B, Timmermans JP, Irobi J, Timmerman V (2013). Charcot-Marie-Tooth causing HSPB1 mutations increase Cdk5-mediated phosphorylation of neurofilaments. Acta Neuropathol.

[47] Selkoe DJ (2001). Alzheimer's disease: genes, proteins, and therapy. Physiol Rev.

[48] Ojha J, Masilamoni G, Dunlap D, Udoff RA, Cashikar AG (2011). Sequestration of toxic oligomers by HspB1 as a cytoprotective mechanism. Mol Cell Biol.

[49] Tóth ME, Szegedi V, Varga E, Juhász G, Horváth J, Borbély E, Csibrány B, Alföldi R, Lénárt N, Penke B (2013). Overexpression of Hsp27 ameliorates symptoms of Alzheimer's disease in APP/PS1 mice. Cell Stress Chaperones.

[50] Baughman HER, Clouser AF, Klevit RE, Nath A (2018). HspB1 and Hsc70 chaperones engage distinct tau species and have different inhibitory effects on amyloid formation. J Biol Chem.

[51] Abisambra JF, Blair LJ, Hill SE, Jones JR, Kraft C, Rogers J, Koren J, Jinwal UK, Lawson L, Johnson AG (2010). Phosphorylation dynamics regulate Hsp27-mediated rescue of neuronal plasticity deficits in tau transgenic mice. J Neurosci.

[52] Cox D, Ecroyd H (2017). The small heat shock proteins αB-crystallin (HSPB5) and Hsp27 (HSPB1) inhibit the intracellular aggregation of α-synuclein. Cell Stress Chaperones.

[53] Cox D, Whiten DR, Brown JWP, Horrocks MH, San Gil R, Dobson CM, Klenerman D, van Oijen AM, Ecroyd H (2018). The small heat shock protein Hsp27 binds α-synuclein fibrils, preventing elongation and cytotoxicity. J Biol Chem.

[54] Shi Y, Jiang X, Zhang L, Pu H, Hu X, Zhang W, Cai W, Gao Y, Leak RK, Keep RF (2017). Endothelium-targeted overexpression of heat shock protein 27 ameliorates blood-brain barrier disruption after ischemic brain injury. Proc Natl Acad Sci USA.

[55] Leak RK, Zhang L, Stetler RA, Weng Z, Li P, Atkins GB, Gao Y, Chen J (2013). HSP27 protects the blood-brain barrier against ischemia-induced loss of integrity. CNS Neurol Disord Drug Targets.

[56] Bidmon HJ, Görg B, Palomero-Gallagher N, Behne F, Lahl R, Pannek HW, Speckmann EJ, Zilles K (2004). Heat shock protein-27 is upregulated in the temporal cortex of patients with epilepsy. Epilepsia.

[57] Iadecola C (2004). Neurovascular regulation in the normal brain and in Alzheimer's disease. Nat Rev Neurosci.

[58] Zhao M, Jiang XF, Zhang HQ, Sun JH, Pei H, Ma LN, Cao Y, Li H (2021). Interactions between glial cells and the blood-brain barrier and their role in Alzheimer's disease. Ageing Res Rev.

[59] Lee H, Pienaar IS (2014). Disruption of the blood-brain barrier in Parkinson's disease: curse or route to a cure?. Front Biosci (Landmark Ed).

[60] Ortiz GG, Pacheco-Moisés FP, Macías-Islas M, Flores-Alvarado LJ, Mireles-Ramírez MA, González-Renovato ED, Hernández-Navarro VE, Sánchez-López AL, Alatorre-Jiménez MA (2014). Role of the blood-brain barrier in multiple sclerosis. Arch Med Res.

[61] Blennow K, Wallin A, Fredman P, Karlsson I, Gottfries CG, Svennerholm L (1990). Blood-brain barrier disturbance in patients with Alzheimer's disease is related to vascular factors. Acta Neurol Scand.

[62] Lee M, Bard F, Johnson-Wood K, Lee C, Hu K, Griffith SG, Black RS, Schenk D, Seubert P (2005). Abeta42 immunization in Alzheimer's disease generates Abeta N-terminal antibodies. Ann Neurol.

[63] Suzuki M, Tezuka K, Handa T, Sato R, Takeuchi H, Takao M, Tano M, Uchida Y (2022). Upregulation of ribosome complexes at the blood-brain barrier in Alzheimer's disease patients. J Cereb Blood Flow Metab.

[64] Awad D, Prattes M, Kofler L, Rössler I, Loibl M, Pertl M, Zisser G, Wolinski H, Pertschy B, Bergler H (2019). Inhibiting eukaryotic ribosome biogenesis. BMC Biol.

[65] Mu F, Chen X, Du X, Jiao Q, Bi M, Jiang H (2021). Regulatory mechanism of interferon regulatory factor 1 by α-synuclein in mouse Parkinson's disease model. Nan Fang Yi Ke Da Xue Xue Bao.

