# Supplementary material for: Integrating single-nucleus sequence profiling to reveal the transcriptional dynamics of Alzheimer’s disease, Parkinson’s disease, and multiple sclerosis
Source: J Transl Med. 2023 Sep 21;21:649. doi: 10.1186/s12967-023-04516-6 (PMC10515258; doi:10.1186/s12967-023-04516-6)
Supplement: Supplementary file 1 — Additional file 1: Fig. S1. The UMAP plots of the expression patterns of the cell markers. [file 12967_2023_4516_MOESM1_ESM.pdf]

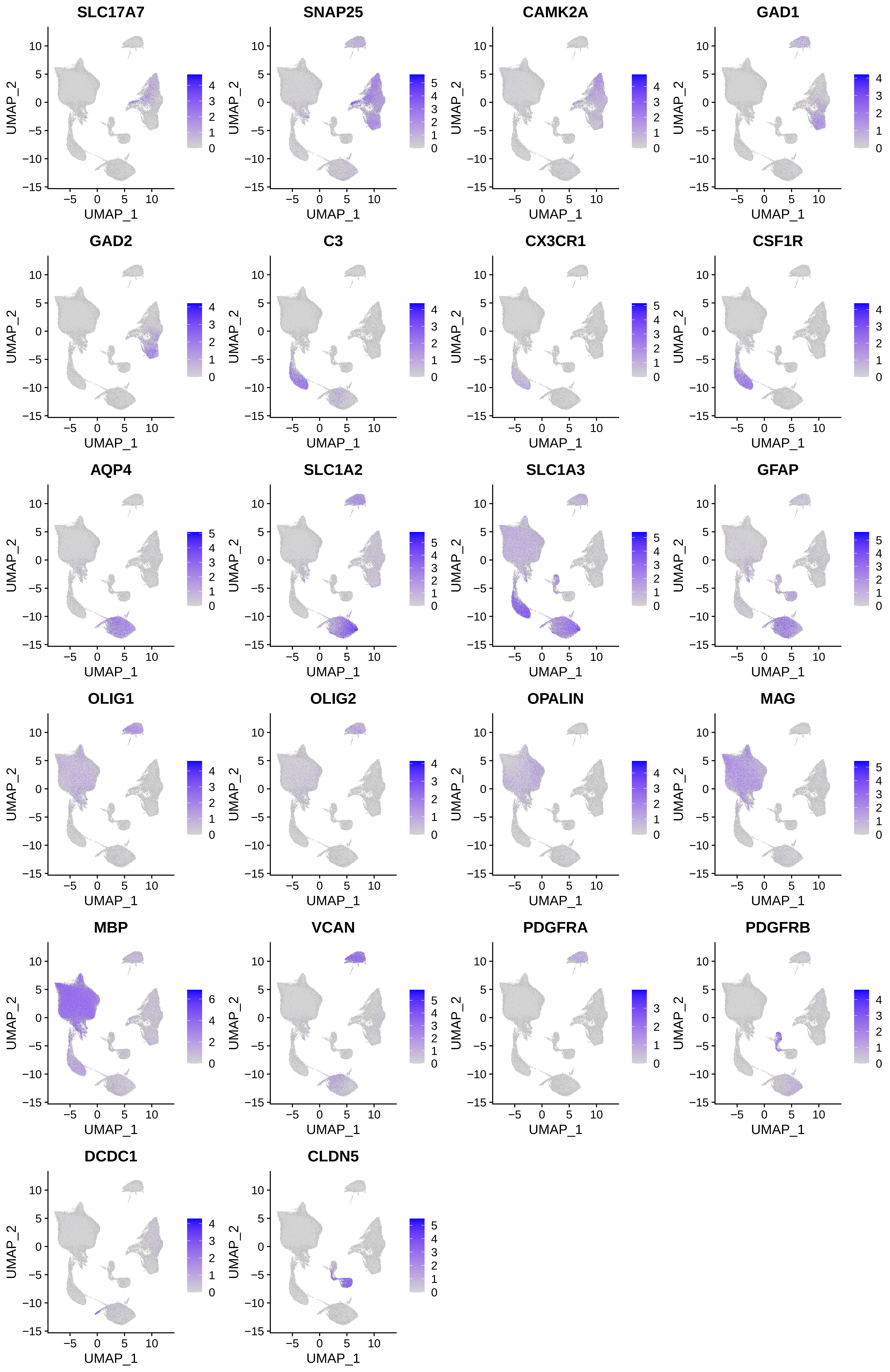

SLC17A7
SNAP25
CAMK2A
GAD1
GAD2
C3
CX3CR1
CSF1R
AQP4
SLC1A2
SLC1A3
GFAP
OLIG1
OLIG2
OPALIN
MAG
MBP
VCAN
PDGFRA
PDGFRB
DCDC1
CLDN5
UMAP_1
UMAP_2
